# The Salivary Secretome of the Tsetse Fly *Glossina pallidipes* (Diptera: Glossinidae) Infected by Salivary Gland Hypertrophy Virus

**DOI:** 10.1371/journal.pntd.0001371

**Published:** 2011-11-22

**Authors:** Henry M. Kariithi, Ikbal A. Ince, Sjef Boeren, Adly M. M. Abd-Alla, Andrew G. Parker, Serap Aksoy, Just M. Vlak, Monique M. van Oers

**Affiliations:** 1 Laboratory of Virology, Wageningen University, Wageningen, The Netherlands; 2 Insect Pest Control Laboratory, Programme of Nuclear Techniques in Food and Agriculture, International Atomic Energy Agency, Vienna, Austria; 3 Laboratory of Biochemistry, Wageningen University, Wageningen, The Netherlands; 4 Department of Genetics and Bioengineering, Yeditepe University, Istanbul, Turkey; 5 Yale School of Public Health, New Haven, Connecticut, United States of America; National Institute of Allergy and Infectious Diseases, United States of America

## Abstract

**Background:**

The competence of the tsetse fly *Glossina pallidipes* (Diptera; Glossinidae) to acquire salivary gland hypertrophy virus (SGHV), to support virus replication and successfully transmit the virus depends on complex interactions between *Glossina* and SGHV macromolecules. Critical requisites to SGHV transmission are its replication and secretion of mature virions into the fly's salivary gland (SG) lumen. However, secretion of host proteins is of equal importance for successful transmission and requires cataloging of *G. pallidipes* secretome proteins from hypertrophied and non-hypertrophied SGs.

**Methodology/Principal Findings:**

After electrophoretic profiling and in-gel trypsin digestion, saliva proteins were analyzed by nano-LC-MS/MS. MaxQuant/Andromeda search of the MS data against the non-redundant (nr) GenBank database and a *G. morsitans morsitans* SG EST database, yielded a total of 521 hits, 31 of which were SGHV-encoded. On a false discovery rate limit of 1% and detection threshold of least 2 unique peptides per protein, the analysis resulted in 292 *Glossina* and 25 SGHV MS-supported proteins. When annotated by the Blast2GO suite, at least one gene ontology (GO) term could be assigned to 89.9% (285/317) of the detected proteins. Five (∼1.8%) *Glossina* and three (∼12%) SGHV proteins remained without a predicted function after blast searches against the nr database. Sixty-five of the 292 detected *Glossina* proteins contained an N-terminal signal/secretion peptide sequence. Eight of the SGHV proteins were predicted to be non-structural (NS), and fourteen are known structural (VP) proteins.

**Conclusions/Significance:**

SGHV alters the protein expression pattern in *Glossina*. The *G. pallidipes* SG secretome encompasses a spectrum of proteins that may be required during the SGHV infection cycle. These detected proteins have putative interactions with at least 21 of the 25 SGHV-encoded proteins. Our findings opens venues for developing novel SGHV mitigation strategies to block SGHV infections in tsetse production facilities such as using SGHV-specific antibodies and phage display-selected gut epithelia-binding peptides.

## Author Summary

Tsetse fly (Diptera; Glossinidae) transmits two devastating diseases to farmers (human African Trypanosomiasis; HAT) and their livestock (Animal African Trypanosomiasis; AAT) in 37 sub-Saharan African countries. During the rainy seasons, vast areas of fertile, arable land remain uncultivated as farmers flee their homes due to the presence of tsetse. Available drugs against trypanosomiasis are ineffective and difficult to administer. Control of the tsetse vector by Sterile Insect Technique (SIT) has been effective. This method involves repeated release of sterilized males into wild tsetse populations, which compete with wild type males for females. Upon mating, there is no offspring, leading to reduction in tsetse populations and thus relief from trypanosomiasis. The SIT method requires large-scale tsetse rearing to produce sterile males. However, tsetse colony productivity is hampered by infections with the salivary gland hypertrophy virus, which is transmitted via saliva as flies take blood meals during membrane feeding and often leads to colony collapse. Here, we investigated the salivary gland secretome proteins of virus-infected tsetse to broaden our understanding of virus infection, transmission and pathology. By this approach, we obtain insight in tsetse-hytrosavirus interactions and identified potential candidate proteins as targets for developing biotechnological strategies to control viral infections in tsetse colonies.

## Introduction

Tsetse flies (*Glossina* sp.) are found exclusively in sub-Saharan Africa and are efficient vectors of African trypanosomes, causative agents of sleeping sickness in humans and nagana in domesticated animals [1]–[3]. Sleeping sickness is invariably fatal if untreated and, until now, the available drugs for sleeping sickness have been unsatisfactory, some being toxic and all difficult to administer [4], and resistance to drugs is increasing [5]. Hence, the search for novel strategies must continue among which are vector-based strategies [6]. Tsetse control remains the most feasible management technique to combat trypanosomiasis and the application of the sterile insect technique (SIT) within the concept of area-wide integrated insect management (AW-IPM), has had promising successes [7], [8]. This strategy relies heavily on colony mass rearing of flies in contained production facilities. The problem is that the production of some species of tsetse such as *Glossina pallidipes* colonies are vulnerable to infections by a salivary gland hypertrophy virus (SGHV) [9]–[12]; which in a proportion of infected flies leads to hypertrophy (hyperplasia) of the salivary glands (hereafter referred to as SGs) and gonadal lesions. Consequently, fly productivity and fecundity drastically drops, often leading to colony collapse, making colony rearing and SIT applications difficult to implement.

A critical step during SGHV infection of tsetse is the viral replication following ingestion of virus-contaminated blood meals [13]. Although it is yet to be established how long after infection the virus is transmitted, it is likely that a requisite to the transmission of the virus is replication and secretion of the virus into the SG lumen. It is currently unknown whether virus transmission is modified by tsetse saliva that is also deposited at the feeding site to enable the blood feeding process [14], [15]. Further, it is currently unknown how SGHV influences fly gene expression in the SGs or how exactly tsetse immune system defends the fly from the injurious consequences of SGHV infection. To date, the non-redundant (nr) protein database of GeneBank has 156 *Glossina* proteins, 17 of which are annotated as found in the fly's SGs [16]. This is in addition to 8 proteins from a previous limited transcriptome analysis of *G. morsitans morsitans* saliva [17]–[20]. Given that knowledge on the mechanisms behind virus replication and transmission processes remains very limited, further studies are required to characterize the molecular interactions between *Glossina* and its SGHV.

The hypothesis of this study is that the competence of *Glossina* sp. to acquire SGHV and to successfully transmit mature virions to its offspring and to other flies in the colony depends on interactions between *Glossina* and SGHV macromolecules. Hence, the *Glossina* SG secretome must encompass a spectrum of proteins required for all the different facets of the SGHV infection cycle. In this study, we investigated the secretome of hypertrophied and non-hypertrophied SGs of the tsetse fly *Glossina pallidipes* to gain a deeper understanding of the composition and putative roles of the fly's saliva in the *Glossina*-SGHV interactions, aiming particularly at the identification of potential targets for development of virus mitigation strategies in mass rearing facilities of *G. pallidipes*.

## Materials and Methods

### Tsetse fly strain and SGHV detection

Two groups of *G. pallidipes* flies, teneral (i.e. within 24 h post emergence) and ten-day old were obtained from the IAEA Insect Pest Control Laboratory Seibersdorf, Austria. To detect the presence of *Glossina pallidipes* salivary gland hypertrophy virus (GpSGHV) infections in teneral flies, total DNA was extracted from one intermediate excised leg of individual flies using the ZR Genomic DNA kit (Zymo Research, USA). The DNA was amplified by PCR using primers and conditions described previously [21], [22]. Flies with negative PCR results were considered as non-infected (hereafter referred to as non-hypertrophied), while flies with positive PCR results were considered as symptomatically infected. The symptomatically SGHV-infected flies (hereafter referred to as hypertrophied) used in this study were naturally infected during the fly mass-rearing, i.e. they acquired SGHV from their mothers. These naturally-infected flies were used in this study because artificial infection of *Glossina* using SGHV preparations from hypertrophied salivary glands either orally or by injection does not lead to hyperplasia in the same generation (unpublished data). The hyperplasia of the SGs was confirmed microscopically during subsequent dissections. The ten-day old experimental flies were divided into eight groups based on hours post feeding (hereafter referred to as hpf) and whether they were hypertrophied or not. These were groups 1A and B at 0 hpf (non-fed = teneral, non-hypertrophied or hypertrophied), and groups of ten-day old flies at 48 (groups 2A and B), 72 (groups 3A and B) and 96 (groups 4A and B) hpf. Flies in groups 1A and B were dissected immediately after PCR results. The flies in the other groups were given a blood meal and maintained in the insectaria in standard rearing conditions [23] until the dissections of the SGs at the respective hpf.

### Harvesting of fly saliva

Harvesting of saliva was performed by adaptation of previously described methods [13], [17], [24], [25]. Briefly, flies were anesthetized by a cold shock (10 min; 4°C), and dissected. For each group, 10 and 40 pairs of intact SGs were aseptically collected from hypertrophied and non-hypertrophied flies, respectively, in 500 µl ice-cold, sterile PBS (pH 7.4) supplemented with EDTA-free protease inhibitor cocktail (Roche Applied Sciences, Germany). Saliva fluid was allowed to diffuse out of the glands into the buffer for 2.5 h on ice and the buffer was subsequently separated from the SGs by brief centrifugation (500 rpm; 2 min; 4°C). The supernatants (i.e., saliva = diffusate from intact SGs) were filtered (0.45-µm filter) and immediately frozen in 100 µl aliquots at −80°C until further analysis.

### Gel electrophoresis and in-gel digestion

To determine the tsetse SGs protein profiles, the saliva samples were separated using SDS-PAGE (12% acrylamide) [26] for 1.1 cm. The gel was stained with Coomassie Brilliant Blue using the Colloidal Staining Kit (Invitrogen). To prepare the proteins for each of the eight groups described above, equal portions of the central third of each entire gel lane, i.e. the middle section spanning the complete gel lane, and containing the saliva proteins was excised. We selected this middle region of the lanes to avoid contaminations from neighboring lanes. The resultant gel sections were cut into approximately 1 mm^3^ cubes and in-gel protein digestions performed as previously described [27]. Supernatants were transferred to fresh Eppendorf microtubes, and the remaining peptides were extracted by incubating the gel pieces with 5% trifluoroacetic acid (TFA)/H_2_O, followed by 15% acetonitrile (ACN)/1% TFA. The extracts were combined, reduced in volume in a speed vacuum and dissolved in 20 µl of 0.1% formic acid/H_2_O. The peptides resulting from this digestion were analyzed by LTQ-Orbitrap Nano liquid chromatography coupled to electrospray and tandem mass spectrometry (nanoLC-MS/MS) as previously described [28].

### Identification and quantitation of the salivary gland proteins

Raw MS/MS files from the LTQ-Orbitrap were generated by MaxQuant software version 1.1.1.36, supported by Andromeda as the database search engine for peptide identification [28]–[31]. MS/MS spectra were searched against a concatenated *G. m. morsitans* decoy database generated by reversing the protein sequences. The database used for peptide/protein searches was derived from the SGs expressed sequence tag (EST) library available from the International Glossina Genome Initiative (IGGI) (http://old.genedb.org/genedb/glossina/). Protein sequences of common contaminants, e.g. trypsin and keratins were used in MaxQuant's “contaminants.fasta” database. MaxQuant was used with a peptide tolerance of 10 parts per million while all other settings were kept as default with one extra addition of asparagine or glutamine de-amidation as variable modification [32] to allow de-amidated peptides to be used for quantification. Bioinformatic analysis of the MaxQuant/Andromeda Work flow output and the analysis of the abundances of the identified proteins were performed with the Perseus module (available at the MaxQuant suite). We accepted peptides and proteins with a false discovery rate (FDR) of less than 1% and proteins with at least 2 unique peptides.

### Signal peptide prediction and gene ontology term mapping of *G. pallidipes* SG proteins

To suggest putative functions for individual tsetse SGs secretome components, the accepted proteins were inputted into Blast2GO v.2.4.8 (http://www.blast2go.org/) [33] and categorized by molecular function (MF), biological process (BP) and cellular component (CC). Gene Ontology (GO) term mapping was based on sequence similarity to previous GO mapped sequences available in the Uniprot database and by merging GOs identified after the InterProScan searches. Signal secretion peptides were predicted from the results arising from InterProScan searches [34], while potential secretion signal peptide sequences and determination of cleavage site positions predicted using SignalP v. 3.0. (http://www.cbs.dtu.dk/services/SignalP/).

### Structural and functional annotation of SGHV-encoded proteins

To predict the conserved domains of SGHV-encoded proteins, the viral protein sequences were inputted into InterPro suite (http://www.ebi.ac.uk/Tools/pfa/iprscan/). Structural and functional annotations were determined by pasting the single-letter amino acid codes of the proteins into the Sequence Annotated by Structure (SAS) interface (http://www.ebi.ac.uk/thornton-srv/databases/sas/) [35]. Further annotations of the information obtained from the PDB output were performed at the pfam site (http://pfam.sanger.ac.uk/).

## Results

### Strategy

For a comprehensive analysis of the *G. pallidipes* SG proteins, we adopted a four-step strategy. (1) Electrophoretic profiling and identification of tsetse saliva proteins by nanoLC-MS/MS, (2) cataloguing of the MS-supported proteins by gene ontology mapping using Blas2GO suite, (3) confirmation of the presence of N-terminal signal peptide sequences in these proteins by InterProScan and SignalP suites, and (4) prediction of the potential *Glossina*-SGHV interactions by analyzing the proteins expressed in hypertrophied SGs.

### Electrophoretic profiling and proteomic analysis

**Figure 1:**
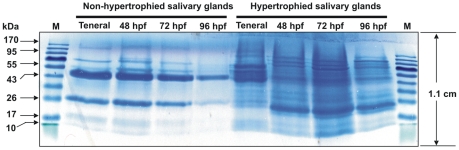
SDS-PAGE profiling of *G. pallidipes* salivary glands proteins. Shown in the Colloidal Blue Stained SDS-PAGE gel are saliva proteins from non-hypertrophied (lanes 2–5) and hypertrophied salivary glands (lanes 6–9) of *G. pallidipes* dissected at 0 hpf (Teneral), 48 hpf, 72 hpf and 96 hpf. Lanes 1 and 10 were loaded with Pre-stained protein marker (Fermentas), for which the sizes are indicated in kDa.

The rationale of using 10 and 40 pairs of intact glands from hypertrophied and non-hypertrophied salivary glands respectively, follows from documented reports that hypertrophied salivary glands are enlarged at least four times their normal thickness [36], [37]. The saliva was harvested from hypertrophied and non-hypertrophied SGs dissected at various time points after feeding as described in the materials and methods and electrophoretic profiles were made (Figure 1). In general, the electrophoretic protein expression profiles detected in the non-hypertrophied and hypertrophied glands correlated well with the viral loads that we have reported in our previous study for these glands [36], [37]. The proteins ranged from <10 kDa to >170 kDa. Visual observation of the SDS-PAGE gel revealed not only clear similarities, but also quantitative differences between the protein profiles of hypertrophied and non-hypertrophied SGs in terms of both proteic band intensities and the presence or absence of several protein bands. Three trends are demonstrated from the protein profiles: (1) A fairly constant protein quantity, albeit a slight decrease at 96 hpf for non-hypertrophied SGs and a maximal quantity at 72 hpf for hypertrophied SGs, (2) A (multiple) high intensity protein band (26 kDa region) in the profile of non-hypertrophied SGs relative to the hypertrophied SGs, and (3) at all the time points, the majority of protein bands in the 19–25 kDa, 29–43 kDa, 55–70 kDa and >95 kDa range present in the secretome of hypertrophied SGs have low abundance or are not detectable in the non-hypertrophied SGs.

A MaxQuant/Andromeda search of LC-MS/MS data against the nr NCBI database was performed for all eight saliva protein samples in the gel lanes (Figure 1). The saliva samples at 72 hpf were arbitrarily chosen for further analysis are they seemed to contain maximum amount of saliva proteins and may also contain the maximum amount of SGHV proteins. This selection criterion is in agreement with previous studies indicating that tsetse saliva production is at peak production 3 days after taking a blood meal [17]. Analysis of these samples by the Perseus module of the MaxQuant suite yielded 521 protein hits, 31 of which were SGHV-encoded based on the known genome and predicted proteome of the virus [28], [38]. Based on an FDR limit of <1% and detection of at least 2 unique peptides per protein (see methods), a further search of *G. m. morsitans* SG EST dataset identified 317 protein hits. Twenty-five of these proteins were SGHV-encoded (Table S1).

### Differential protein expression in hypertrophied and non-hypertrophied SGs

Based on the MS/MS data and the electrophoretic protein profile in Figure 1, a comparison of the protein expression patterns in the non-hypertrophied and hypertrophied SGs within and between the different time points revealed noteworthy findings. Firstly, the relative composition of saliva proteins in the non-hypertrophied SGs remained fairly constant from 0 hpf to 72 h pf, followed by a slight drop at 96 hpf, unlike in the case of the non-hypertrophied SGs (Figure 1). This is in agreement with a previous report by Van Den Abbeele and his group [17]. Secondly, at all the time points, at least 32 *Glossina* proteins are highly up regulated i.e. they are expressed in high abundance in the hypertrophied SGs but were either detected in very low abundance or not detectable at all in the non-hypertrophied SGs (Table 1). Thirdly, some *Glossina* proteins are down regulated in the hypertrophied SGs relative to the non-hypertrophied SGs. Nine of the most down-regulated proteins at all the time points are indicated in Table 2. Taken together, these differential protein expressions between hypertrophied and non-hypertrophied SG implies that infection of *Glossina* by SGHV greatly alters the protein expression pattern in flies with hypertrophied SGs. Lastly, the maximal expression of proteins was found in the hypertrophied SGs at 72 hpf (Figure 1). This agrees well with previous reports that saliva production in *Glossina* reaches maximum 2–3 days after a blood meal [17].

**Table 1:** Thirty-two *G. pallidipes* secretome proteins uniquely expressed in hypertrophied salivary glands.

Mol. wt range	Description of the proteins expressed in high abundance in hypertrophied salivary glands		
	Protein Name	Accession No.	Functional annotation
<26 kDa	Bis-(5′-nucleosyl)-tetraphosphatase	XP_001969373.1	DNA replication, metabolic stress and apoptosis
	Niemann-Pick TypeC-2	ADD20212.1	Mesoderm development
	RNA polymerase II - BTF3	ADD19794.1	Transcription
	deoxyUTP-pyrophosphatase	ADD20717.1	Chromosomal integrity
	Transitional ER ATPase	XP_002016299.1	Molecular chaperones
	ADP-ribosylation factor 1	ADE42873.1	Vesicle coat protein assembly
	DNA replication factor/protein phosphatase inhibitor SET/SPR-2	ADD19576.1	Movement of histones; nucleasome assembly; chromatin fluidity
	GTPase -binding nuclear protein Ran/TC4/gSP1	ADD18812.1	Nuclear transport and DNA replication
	Cu/Zn-superoide dismutase	ADD19264.1	REDOX processes
26–43 kDa	Ras-like GTP-binding protein Rho1	ADD18925.1	Control of cytoskeletal changes
	Proteasome activator complex subunit 3	ADD19370.1	Binds to 20S proteasome
	Aldo/keto reductase	ADD18539.1	Oxidoreductase activities
	26S proteasome non-ATPase regulatory complex	ADD18827.1	Integrity of 26S proteasome complex
	Quiescin-sulfhydryl oxidase4	XP_002048727.1	Localized in high concentrations in cells with heavy secretory load
43–55 kDa	Serine protease inhibitor4	ABC25075.1	Chaperoning; Toll signalling
	60s acidic ribosomal protein P0	ADD19996.1	Proteins synthesis
	S-adenosylmethionine synthetase	ADD19751.1	Supplies metabolic methyl groups by catalyzing synthesis of AdoMet
	ATP-dependent RNA helicase	XP_001968815.1	Splicing and ribosome biogenesis
	Vacuolar ATPase-S1 Ac45	ADD18511.1	REDOX; NAD/NADH-binding
	Imaginal growth factor-3	ABC25095.1	Chitinase-related GH18; interaction with surface glycoproteins
	Hsp-cognate70Cb	CAB38172.2	Protein folding
	Yellow protein precursor	ADD19747.1	Controls adult pigmentation
>55 kDa	Catalase	ADD20421.1	Protection from peroxides
	Hsp70/Hsp90 organizing protein	ADD19147.1	Protein-protein interactions; chaperoning; transcription; protein transport complexes
	Juvenile hormone esterase	ADD18773.1	Hydrolase
	Hsp60 (GroEL chaperonin)	ADD20133.1	Productive protein folding
	Angiotensin-converting-enzyme	XP_002002462.1	Membrane-located metallopeptidase
	Amidophosphoribosyltransferase	XP_002083899.1	Purine biosynthesis
	Lysosomal-α-manosidase	ADD18519.1	Carbohydrate metabolism
	C1-Tetrahydrofolate synthase	ADD18346.1	Carbon metabolism
	Endocytosis/signalling protein EHD1	ADD19069.1	Endocytosis; vesicle transport; signal transduction
	Gamma-glutamyl phosphate reductase	ADD19821.1	L-proline biosynthetic pathway

The thirty-two *G. pallidipes* proteins expressed in high abundance in the hypertrophied SGs at all the time points (0, 48, 72 and 96 hpf) but were either detected in very low abundance or not detectable at all in the non-hypertrophied salivary glands.

**Table 2:** Nine *G. pallidipes* proteins that are down-regulated in hypertrophied salivary glands.

Protein Name	Mol. Wt (kDa)	Accession No.	Description of functional domains
Tsal2 protein precursor	17.541	ADD19043.1	DNA/RNA non-specific endonuclease
5′-nucleotidase family salivary protein	18.229	ADD20435.1	Cellular energy metabolism
Tsal1 protein precursor	22.509	ADD20565.1	DNA/RNA non-specific endonuclease
Larval serum protein 2	23.716	ADD18255.1	store of amino acid for synthesis of adult proteins
Salivary antigen 5 precursor	28.901	ADD18879.1	CAP family protein with unknown function
20S proteasome regulatory-β-type	30.592	ADD19908.1	Central enzyme of non-lysosomal protein degradation
Salivary secreted adenosine	47.831	ADD20094.1	A metallo-dependent hydrolase
Salivary gland growth factor-1	5.388	ADD18584.1	Adenosine deaminase-related growth factor
Apyrase-related protein	53.977	ADD18451.1	Cellular energy metabolism

Summary of nine of the most down regulated proteins in the hypertrophied salivary glands. The proteins are arranged in the order of molecular weights.

**Figure 2:**
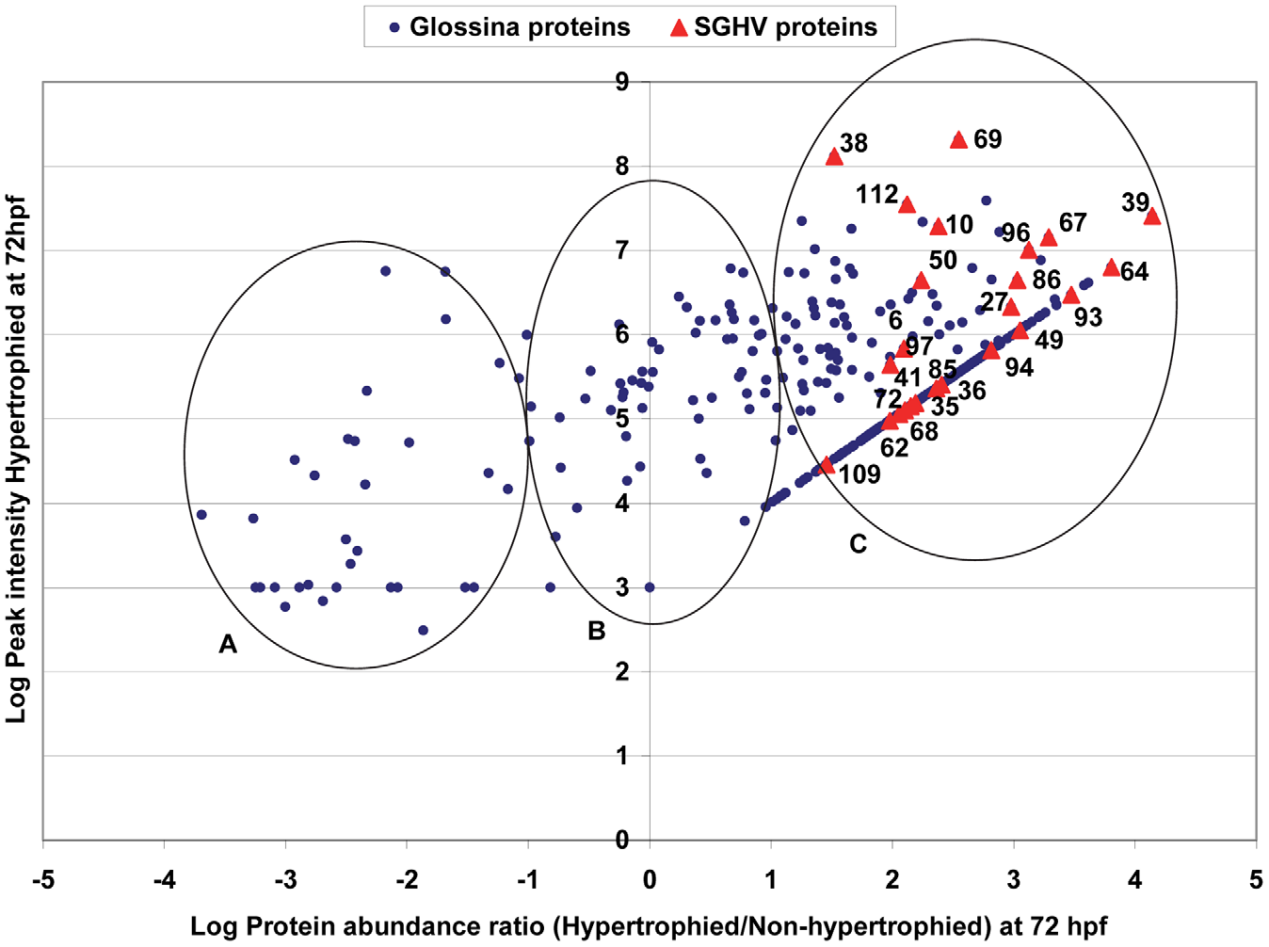
Plot of abundance of *G. pallidipes* (dots) and SGHV (triangles) proteins. The figure shows protein abundance ratios between hypertrophied and non-hypertrophied salivary glands collected at 72 hpf. *G. pallidipes* and SGHV proteins are indicated in blue dots and red triangles respectively. Shown are the most abundantly expressed proteins (group A), the least abundant (group B) and the proteins detected in the hypertrophied salivary glands but were not detectable in the non-hypertrophied salivary glands (group C). To determine the ratio for group C the value for the non-detectable proteins was set to 1000. This value is just below the lowest value obtained for the least abundant protein (cn8877 Salivary gland growth factor-2).

**Figure 3:**
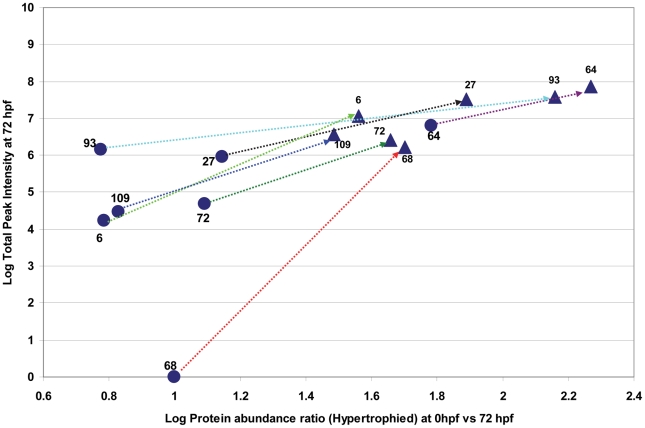
The shift in abundance of SGHV proteins from 0 hpf to 72 hpf. The figure shows the shift observed in the expression of 7 SGHV proteins detected in the secretome of hypertrophied salivary glands of *G. pallidipes* dissected at 72 hpf (circles) relative to 0 hpf (triangles).

Based on the analysis of the differential protein expression patterns, we focused on the abundances of saliva proteins from hypertrophied and non-hypertrophied SGs at 72 hpf relative to 0 hpf in order to investigate perturbations of the protein expression in the fly SGs. We first compared *Glossina* and SGHV protein abundance ratios between hypertrophied and non-hypertrophied SGs (Figure 2) to find the really significance differences between the two samples. Three abundance patterns could be deduced. First, 39.4% (115/292) of *Glossina* and 52% (13/25) SGHV proteins were abundantly expressed in hypertrophied flies relative to non-hypertrophied flies (Figure 2A). Second, 14.4% (42/292) of *Glossina* proteins showed relatively low abundance regardless of SGHV infection (Figure 2B). Lastly, 46.2% (135/292) and 48% (12/25) of *Glossina* and SGHV proteins were specifically expressed in the hypertrophied SGs, respectively (Figure 2C). A plot of the shift in the abundance of a selection of seven SGHV proteins uniquely expressed in hypertrophied flies at 72 hpf relative to teneral flies is shown in Figure 3. The host proteins abundantly expressed in hypertrophied SGs at 72 hpf showed a similar trend (data not show). These uniquely expressed proteins at 72 hpf have implications in the *Glossina*-SGHV molecular interactions (see discussion).

### Gene ontology and signal peptide sequence mapping

Secondly, we conducted gene ontology (GO) mapping on the saliva proteins. At least one GO term could be assigned to 285 of the 317 detected saliva proteins. Five (1.8%) *Glossina* proteins were deemed of unknown function after blast searches against the nr databases and annotation augmentation. The GO terms assigned to individual *Glossina* proteins are shown in Table S1, while the terms assigned to SGHV proteins are shown in Table S2.

**Figure 4:**
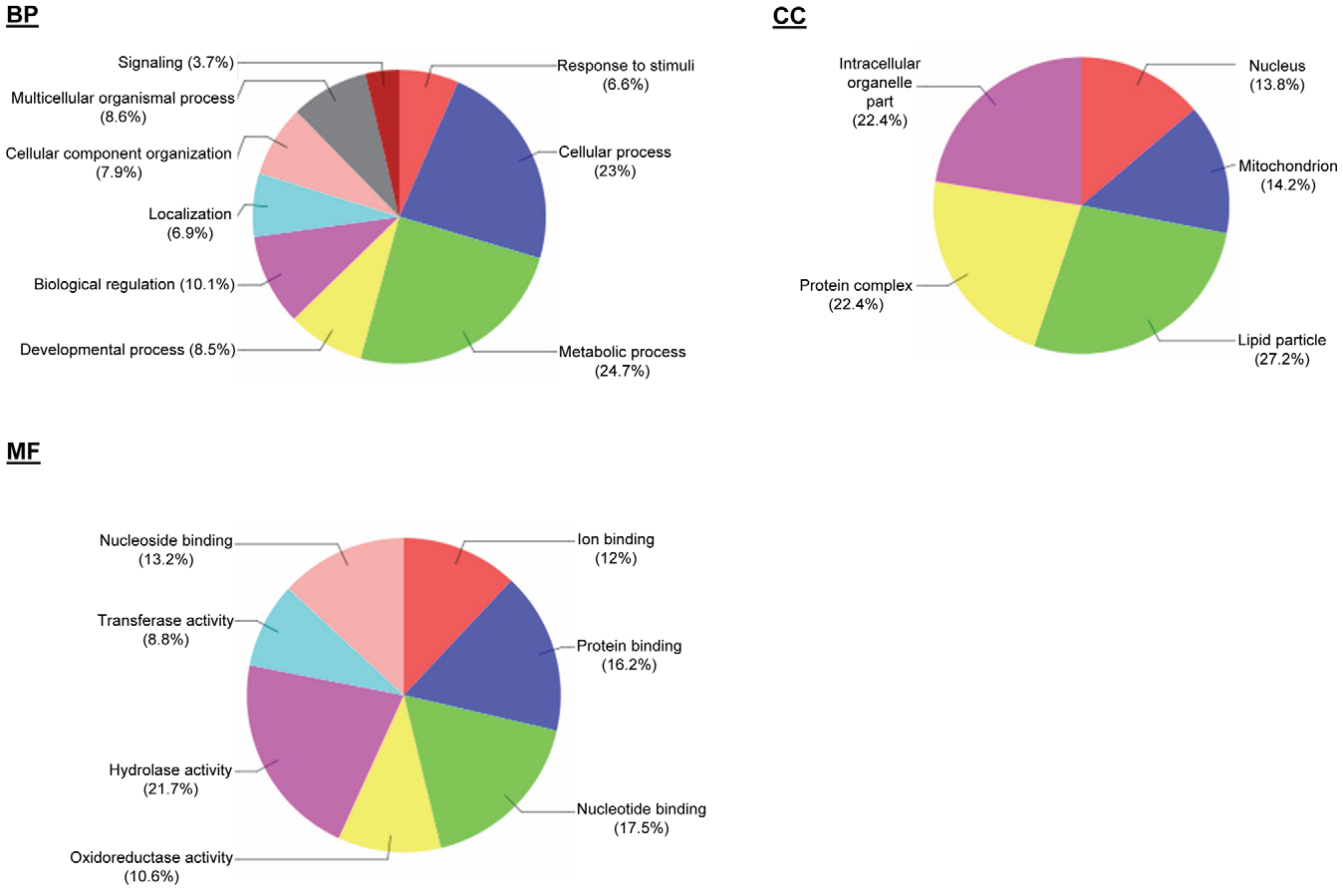
Annotation of *G. pallidipes* salivary glands secretome proteins. Gene Ontology (GO) terms for categorization of *G. pallidipes* salivary gland secretome proteins by molecular function (MF), biological process (BP) and cellular component (CC) to suggest the functional components of the secretome.

Based on the GO annotations, the MS/MS-supported proteins were grouped into three categories: (1) the broad biological processes (BP) the proteins are involved in, (2) the predicted molecular functions (MF) they perform, and (3) the sub-cellular structures/locations/macromolecular complexes or components (CC) these proteins associate with (Figure 4). The three most common BPs were metabolic processes (24.7%), cellular processes (23%) and biological regulation (10.1%) whereas the three most common MFs were nucleotide/nucleoside-binding (combined percentage of 30.7%), hydrolase activity (21.7%), protein binding (16.2%) and transferase activity (13.2%). The multilevel CC analysis returned five sub-categories, of which the highest proportion (27.2%) of the proteins showed association with lipid-related metabolism (sub-categories of CC, Figure 4). These observations are not entirely unexpected. Other studies have shown considerable changes in cellular organization and metabolism in diseased insects. For instance, during the infection of mosquitoes by densonucleosis (DNV) and crane fly by *Tipula* iridescent (TIV) viruses, an enlarged nucleus in target cells was noted to be accompanied by a large increase in nuclear DNA synthesis and a massive enlargement of fat body cells [39]. We also noted that a relatively high proportion (23%) of the identified tsetse SG proteins is associated with cellular processes (BP). Although a full explanation for this observation remains to be confirmed, cellular modifications would be expected in SGHV-infected *Glossina* SG cells, in terms of the formation of vesicles and/or multivesicular bodies associated with various organelles such as the endoplasmic reticulum. Indeed, Sang *et al.*, showed that the cytoplasm of SGHV-infected cells of *G. m. centralis* are heavily vacuolated, show complete disintegration of cytoplasmic organelles, including the smooth and rough ER and the mitochondria, leaving the nuclei scattered around [40]. These membrane and organelle changes could also be involved in processes such as viral mRNA translation, assembly of protein complexes of both SGHV and *Glossina* origin, as well as cell-to-cell transport.

Lastly, we used the InterProScan suite to determine which of the identified saliva proteins contained predicted secretion signals. This analysis revealed that 96.5% (282/292) of *Glossina* and (6/25) and 24% of SGHV MS/MS-supported proteins contained predicted secretion signals, respectively. Further, SignalP analysis of these proteins confirmed that 23% (65/282) of these *Glossina* proteins and the 6 SGHV proteins contained N-terminal signal peptide sequences. Table S1 shows the 65 *G. pallidipes* saliva proteins with signal peptides predicted by SignalP (hereafter referred to as the SG secretome proteins). Structural and functional annotation of all detected SGHV-encoded proteins revealed that 14 were non-structural (NS) an 11 were structural/capsid (VP) proteins, respectively (Table S2).

It is noteworthy that some of the detected *Glossina* proteins may not be synthesized in the SGs. For this, alternative mechanisms for the translocation of proteins into SGs should be taken into account. We expected *Glossina* to have a large number of saliva effector macromolecules with complex effects due to their unique blood-sucking nature. Perhaps some of the identified proteins and macromolecules are synthesized in other organs and then transported into the SGs through hemolymph, by yet-to-be identified mechanisms. This is likely because SGs are capable of sequestering proteins from the hemolymph and then secreting them [41]–[43].

## Discussion

### Differential protein expression in hypertrophied and non-hypertrophied SGs

Functional analysis of the *Glossina* proteins that are abundantly expressed in the hypertrophied SGs but are either not detectable or are expressed in very low abundance in the non-hypertrophied SGs revealed that approximately 31% (10/32) may be involved in DNA replication (see Table 1). These uniquely expressed proteins also include molecular chaperones (transitional ER ATPase), and proteins involved in regulation of signalling (serine protease inhibitor 4), and protein-protein interactions (Hsp70/Hsp90 organizing protein) productive protein folding (Hsp60 or GroEL_like chaperonin protein). Other noteworthy proteins include quiescin-sulfylhydryl oxidase 4, a protein usually localized in high concentrations in cells with heavy secretory loads, Ras-like GTP binding protein (Rho1) which is involved in the control of cytoskeletal changes, and imaginal disc growth factor 3, a chitinase-related GH18 protein involved in the interactions with surface glycoproteins.

Some of the down-regulated proteins such as tsetse salivary gland proteins 1 and 2 (Tsal1/2) and tsetse antigen 5 protein are of unknown function. It is possible that the need for bulk synthesis of proteins for maturation of the virions in hypertrophied SGs could have led to the down regulation of the larval serum protein-2, which serves as a store of amino acid for synthesis of adult proteins. Additionally, the down regulation of salivary apyrase, which has been postulated to facilitate blood location and blood feeding [44], is understandable because flies with hypertrophied SGs almost stop feeding 10–15 days post emergence (unpublished data). Overall, the up-regulation of these proteins, coupled to the down-regulation of the proteins described in Table 2 signifies the alteration of protein expression in hypertrophied *Glossina* SGs as SGHV hijacks key molecular processes as discussed later in this article.

Our approach in establishing the *Glossina* SG secretome was based on the assumption that the SG proteins would have N-terminal signal sequences. Two clear trends were inferred from the cataloguing of saliva proteins in this study: (1) a high proportion of *Glossina* SG secretome proteins indeed have signal peptide sequences, and (2) only a low proportion of the secreted proteins associated with *Glossina* saliva (∼4.9%) are of unknown function. The percentage of non-annotatable *Glossina* SG secretome proteins is substantially lower than the average of non-annotatable proteins across other systems such as the pea aphid *Acyrthosiphon pisum* genome (30%) [45]. This high percentage of annotable proteins in tsetse SGs is probably due to the highly specific function of *Glossina* saliva in blood feeding.

It is likely that our stringent analysis and assumption that the secretome proteins must have N-terminal sequences to substantiate their inclusion into the *Glossina* secretome may have excluded some important proteins from our secretome pool. These proteins do not necessarily need to be excluded from being part of the *Glossina* secretome. As this is the first study of the secretome of the *Glossina* SGs, there is insufficient data available to provide insights into the roles of a majority of the identified proteins. However, potential effector roles of many of these candidate proteins, particularly in the interactions between *Glossina* and SGHV macromolecules, can be predicted based on their homology or similarity to other proteins involved in vector-virus interactions. In this regard, we discuss a selection of the secretome proteins in the context of six categories of functional proteins, and in relation to SGHV infection.

### Protein folding machinery

A high abundance of heat shock proteins (HSPs) was identified in the *Glossina* hypertrophied SG secretome. HSP induction by viruses is not the result of a meager “canonical” HSP-mediated heat shock response (HSR), but rather the effect of virus-controlled transcriptional/translational switches, sometimes involving individual viral products [46]. Our data present strong indication of hijacking of the host cell chaperon machinery for correct folding of abundant SGHV proteins rapidly synthesized in bulk, and for their correct assembly into viral components during the different phases of viral replication. These observations have been documented in previous studies (see [47]–[49] for review). For instance, we detected Torsin-like-protein-precursor in hypertrophied SGs as opposed to the non-hypertrophied SGs. This protein is localized in the endoplasmic reticulum (ER) lumen and is involved in unfolded protein binding as well as chaperone-mediated protein folding [50], [51].

Many viruses interact with HSPs at different infection stages. In Epstein-Barr Virus (EBV), virus attachment at cell membrane receptors activates signal transduction pathways interfering with the heat shock response (HR) [52]. Surface exposed hsc70 and hsp70 proteins are involved in virus entry into cells [53]–[55] and hsc70 may play an active role in virus entry into the host cell as well as at a post-attachment step [56]. In addition, hsp70 chaperones are involved in disassembly of oligomeric protein structures and viral internalization into host cells (see review in [57]–[63]). Viral proteins, including *E1A* of Adenovirus [64], large T antigen (*T* ag) of Simian vacuolating virus 40 [65], [66], ICP4 of Human simplex virus (HSV-1), *IE2* of human cytomegalovirus, and nuclear antigen 3 (*EBNA3*) of EBV [67]–[72], modulate *hsp*70 by direct interaction with different components of the basal transcription apparatus. Additionally HSP70-cognate 4 (HSP70-4) plays an important role in the homeostasis and suppression of O'nyong-nyong virus (ONNV) replication and in the establishment of latent infections in the mosquito *Anopheles gambiae* [73]. It is noteworthy that HSP70-4 was detected in the secretome of hypertrophied SG, as opposed to the non-hypertrophied SGs. Although it remains to be established in the case of SGHV infection of *Glossina*, this protein may play roles in the establishment of asymptomatic SGHV infections in *Glossina* [13].

### Pathogen recognition and defense response proteins

We also identified some members of inducible secreted polypeptides, including C-type lectins (CTLs) (Table S1). CTLs play important roles in insect defense by recognizing pathogen-associated molecular patterns (PAMPS) [74], [75] in invading pathogens [76], [77]. The expression of lectins during virus infection would be expected because they have been shown to mediate immune functions [78], including activation of the lectin complement pathway that also exists in arthropods, thus binding to carbohydrates expressed on viral glycoproteins. In this regard, we detected members of thioester-containing proteins (TEPs) (Table S1) which have been described in the complement system of *Drosophila melanogaster* and *An. gambiae* [79]. It is therefore likely that the hypertrophied SGs express CTLs to block glycoprotein-mediated attachment of SGHV to non-infected SG cells. Although CTLs generally diffuse during SGHV infection, their binding to circulating virus could effectively reduce viral infection of lectin-expressing SG cells. Binding of CTLs to virus and subsequent deposition of complement components on the virus membrane can also lead to enhanced infection of cells that express complement receptors.

### Protein export machinery

ADP-ribosylation factor (ARF) is an abundant protein that reversibly associates with Golgi membranes, and is implicated in the regulation of membrane traffic through the secretory pathway [80]–[82]. This pathway is important for processing of viral contents into complexes capable of nuclear penetration. ARFs have been shown to be up-regulated and involved in virus infection [83]–[85], and possibly explains the detection of ARF in the hypertrophied SGs at 48, 72 and 96 hpf, as opposed to non-hypertrophied and teneral-hypertrophied SGs at 0 hpf. The data presented here indicate the recruitment/hijacking of ARFs to membranes by SGHV and may provide clues for future identification of the pathways utilized by the virus in the replication process in hypertrophied SG.

### Proteases and protease inhibitors

Six types of serine proteases were detected in the secretome of hypertrophied SG but not in the non-hypertrophied SG. Additionally, a precursor of phosphenol oxidase activating factor of the prophenol-oxidase-activating system (proPO-AS), an important component of the innate immune response in insects [86]–[88], was also detected. These two proteins are involved in initiating a signal cascade that eventually leads to melanization reaction which includes the formation of toxic intermediary compounds to kill invading viruses [87]. Studies have shown that the baculovirus P74 is a viral attachment protein [89]–[91]. Cleavage of P74 by trypsin is crucial for infection, probably by to exposing a receptor binding domain [92], enabling interaction with host receptors. SGHV P74 was not detected in this study, which is probably due to its low abundance as demonstrated in our previous study [28]. The expression of the serine proteases in hypertrophied SG is probably required for the interaction of SGHV with the host SG cells receptors as has been reported for several entomopathogens [93], [94].

### Housekeeping genes

RNA polymerase II general transcription factor (BTF3), the translation elongation factor EF-1 gamma (EF1γ), and the ATP-dependent RNA helicase were among the factors detected in the secretome of hypertrophied SG. BTF3 is a general transcription factor necessary for activation of a number of viral promoters by RNAP II [95], while EF1γ is involved in the regulation of protein assembly and folding [96], [97]. The detection of these proteins in hypertrophied SGs, coupled to the presence of RNA helicase is desirable for the expression of replication- and maturation-related genes. In addition, proteins involved in signal transduction were also detected (in SGs dissected 48, 72 and 96 hpf) including GTPase-activating protein (GAP), cAMP-dependent protein kinase, and Ras-related small GTPase (Rho type). GAP is known to be necessary for efficient virus infection and replication [98], and is implicated in the regulation of anterograde traffic between the ER and the Golgi complex, while cAMP-dependent protein kinase is implicated in the regulation of virus infection and virus-induced cell-cell fusion.

### Cytoskeletal proteins

Most viruses use components of the host cytoskeleton to move within cells. Upon virus infection, virions or sub-viral nucleoprotein complexes are transported from the cell surface to the site of viral transcription and replication. During viral escape, particles containing proteins and nucleic acids move again from the site of their synthesis to that of virus assembly and further to the plasma membrane [99], [100]. Viral (sub) particles, particularly in members of herpesviridae, adenoviridae, parvoviridae, poxviridae and baculoviridae use the microtubule and the actin cytoskeleton. In this study, actin 5C, actin 87E and actin depolymerizing factor were detected in all saliva samples except in the non-hypertrophied SG collected 96 hpf. F-actin capping protein was detected in the saliva of hypertrophied SGs 48 hpf, while actin 57B was detected, albeit in low abundance, in hypertrophied SGs 72 hpf. Myosin heavy chain, which drives transport along actin filaments [99] was detected in the saliva of hypertrophied SGs except at 96 hpf, which probably indicates reduced active transport of SGHV virions at this time point. While all the 6 cytoskeletal proteins were detected in the SGs 96 hpf, none were detected in the secretome of non-hypertrophied SGs 96 hpf. It is possible that this could be due to the hyperplasia of the SGs by SGHV, which could potentially lead to lysis of SG cells during advanced stages of viral infection. Further studies are required to investigate these observations.

### Functional annotation of SGHV- proteins detected in the saliva of *G. pallidipes*

The movement and/or replication of viruses in insect vectors require specific interactions between viruses and host components. DNA viruses have evolved mechanisms to evade the host restrictions at entry, cytoplasmic transport, replication, protein synthesis, innate (and for mammalian viruses, adaptive immune) recognition, and egress from the infected cells. The SGHV genome is a circular double-stranded (ds) DNA molecule [38]. Nuclear-replicating viruses with ds DNA genomes such as herpesvirus engage in all aspects of cellular metabolism [101]. Although it has yet to be established for SGHV, DNA viruses adopt the host transcriptional apparatus and all cellular pathways required for processing and transport of their mRNAs. The host cellular mechanisms translate and turn over viral proteins, while transport of viral macromolecules takes place through cellular organelles and structures. In this study, structural and functional annotation of the identified SGHV proteins also indicate their engagement with *Glossina* cellular metabolism. In the following sections, we briefly discuss potential roles played by these SGHV proteins and their possible interactions with *Glossina* SG secretome proteins during the different facets of the viral infection cycle. Inferences below are drawn from other nuclear-replicating DNA viruses, including adenoviruses, hepadnaviruses, herpesviruses, papillomaviruses, and polyomaviruses.

### SGHV entry into host cells

The stepwise entry of DNA viruses into host cells requires viral attachment to cell surface receptors and lateral movements of the virus-receptor complex to specialized sites on the plasma membrane [102]–[105]. In closely-related baculoviruses, *per os* infectivity factor proteins (PIFs) have been shown to be involved in viral attachment to the host cells. [38], [106]–[110]. In the current study, PIFs had very low abundance as was also noted in our previous proteomics study of GpSGHV [28]. In this study, SGHV085 was annotated to be a tyrosine kinase-dependent signaling structural protein, and is probably involved in transport of SGHV polypeptide into the nucleus (see next section) [111]. Additionally, viruses in general elicit signals following attachment to the host cell membrane to circumvent the host defense mechanism. In this regard, annotation revealed SGHV046 as a glutathione S-transferase-like protein, thus pointing to its involvement in this type of signaling.

### Bidirectional cytoplasmic transport of SGHV particles to the nucleus

DNA viruses that replicate their genomes in the nucleus use microtubule motors for trafficking towards the nucleus and the periphery during egress after replication [111]–[113]. Bidirectional transport allows precise delivery of capsids to ensure nuclear targeting, and has been demonstrated in HSV-1) [114], [115] and in human adenovirus 2/5 (Ad2/5) [116]–[118]. Incoming DNA viruses expose proteins on the capsid that preferentially recruit microtubule motor complexes [112], and may release tegument proteins before they traffic to the nucleus [119]. To regulate capsid transport, protein phosphorylation by viral and/or host cellular kinases modulate tegument protein composition as in the case of vaccinia virus [120]. In this study, cAMP-dependent protein kinase detected in the SG secretome is probably involved in anterograde trafficking of SGHV. Additionally, the SGHV041 protein detected here is a Casein kinase (isoform-δ), which is likely to be involved in phosphorylating cytoskeletal components both in anterograde and egress [121], [122]. Early during infection, some viruses such as δ-2 human herpes virus 8 and hepatitis C virus induce Rho GTPases [98], [123], which alter the dynamics by increasing the acetylation of actin microfilaments thereby enhancing viral capsid trafficking transport to the nucleus and establishment of successful infection. Again, Ras-related small GTPase (Rho type) as well as GTPase-activating protein (GAP) were detected in the *Glossina* hypertrophied SGs, and suggest their participation in viral trafficking towards the nucleus. Finally, although the role of spectrins in cytoplasmic transport is not clear, this study identified SGHV010 to have spectrin repeat domains, indicating its potential involvement in SGHV anterograde trafficking.

### Docking, uncoating/disassembly and release of SGHV DNA into nucleus

Cytoplasmic transport is followed by viral genome docking and uncoating at the nuclear pore complex (NPC), a stepwise programme involving partial proteome degradation of incoming capsid or tegument proteins [124], [125]. Although it is not clear how uncoating at the NPC occurs, experiments with some viruses such as herpes B virus have indicated that capsids are transported to the nuclear membrane where they bind to NPCs and release their genome into the nucleus [126]. Additionally, cytoplasmic processing of incoming capsids makes them competent for docking to the NPC [114], and probably prevents the naked viral chromatin from traveling through the cytoplasm, which could trigger DNA-sensing host innate immune responses as has been demonstrated in adenovirus [127]. The SGHV006 protein detected in this study was determined to have α/β-hydrolase catalytic domain, a signature domain for lecithin∶cholesterol acyltransferase (LACT) which is involved in membrane docking of viruses to NPC, as well as in nucleocytoplasmic transport of capsids (see [114], [128]–[131] for review).

### Development of SGHV transcription and replication

Upon infection, some viruses such as *Autographa californica* multicapsid nucleopolyhedrovirus (AcMNPV) establish centers for transcription, DNA replication and progeny nucleocapsid assembly [101], and others express at least one regulatory protein that interacts directly with similar domains such as the promyelocytic leukemia protein nuclear bodies (PML-NBs) [101]. In this study, SGHV112 annotation revealed the presence of the helix-turn-helix characteristic domains of regulatory proteins [132] involved in DNA-protein interactions. Gamma-interferons were detected in this study in the hypertrophied SG dissected as early as 48 hpf, as well as a 19.3 kDa *Glossina* protein encoded by GMsg-6444 (a SUMMO-binding protein). The ubiquitin-like protein SUMO is a partner protein to viral replication center and are dramatically enhanced by interferons [133]. Viral proteins associating with these centers have the ability to stimulate lytic infection and induction of reaction from quiescence [134]. This supports observations that majority of SGHV infections in tsetse colonies are in fact asymptomatic [13], and that there is virus induction in infected flies after an initial blood meal (unpublished data). Also detected in this study were SGHV035 and SGHV036, homologs of thymidylate synthase and deoxycytidylate hedroxymethylase, respectively. The former is involved in regulating a balanced supply of dNTPs during DNA replication [135], [136], while the latter is involved in pyrimidine metabolism [137], [138]. Further, our annotation predicted that SGHV039 (a HSP90-like ATPase) is possibly involved in regulation of unwinding of DNA supercoil strands. Additionally, SGHV062, a p53 transcription factor-like protein containing β-sandwich domain of the sec23/24 superfamily was detected. Proteins of this family are involved in chromosomal segregation and are induced early during the S-phase of the cell cycle [139]–[142], and hence have direct roles in viral DNA transcription and replication. Further, detected in this study was an ABC ATPase-like family protein (SGHV064). Studies have implicated members of this protein family to be involved in translation initiation, ribosome biosynthesis and virus capsid assembly of other viruses such as HIV [143], [144]. Taken together, the presence of these *Glossina* and SGHV proteins in the hypertrophied SGs are an indication that SGHV genome associates with the periphery of PML-NBs, and that viral replication compartments would develop from these sites, as has been observed in other viral systems for instance in HSV-1 [145], [146].

### SGHV maturation and nuclear egress

SGHV049 detected in this study was predicted to be a pre-mRNA splicing factor-9-like protein. The WD40/G-β-repeats represent in this protein is a signature domain for proteins that associate with the spliceosome [147]). Other detected proteins that have potential roles in SGHV maturation were: (1) SGHV072, a FAD-dependent sulfhydryl oxidase (with a late promoter motif and hence likely to be involved in virion maturation, [148]); (2) SGHV093, an uncharacterized endonuclease type-IIe-like protein with DNA-binding and cleavage activities [149]; (3) SGHV027, a chitinase-II (*O*-glycosyl hydrolase) protein, which like the chitinases family-18 proteins may be involved in virus maturation (see [150]–[152] for review); (4) SGHV038, a protein containing α-β-barrel active site, thus likely to be involved in the expression of receptor proteins for membrane transport (egress) [153] and (5) SGHV096, a metal (Mn^2+^) and ion (S_2_O_4_^2−^)-binding protein with multiple TMs, thus likely to be part of SGHV ion-channel proteins. Newly assembled enveloped viruses recruit periphery directed motors, and are transported to the plasma membrane on the microtubules upon binding of the outer membrane [113] proteins and fuse with plasma membrane. With involvement of host tyrosine kinases [112], the virus may eventually be launched away from the infected cell and spread the viral progeny. Although it is unclear whether SGHV travel in vesicles or as capsid as baculoviruses do, SGHV097 was predicted to be a vesicle-associated membrane protein and could be involved in targeting and/or fusion of virus-containing vesicles to the target membranes [119], [154].

### Conclusions

The *Glossina pallidipes* species is found in several African countries and rearing facilities have been established in Kenya, Ethiopia and Tanzania with the aim of strengthening the fly eradication campaigns in African. Salivary gland hypertrophy virus infection leads to drastic negative effects on the productivity and stability of the *G. pallidipes* colonies and in certain cases to colony collapse. In addition, a recent study [155] has demonstrated that this virus is widely distributed in the wild populations of *G. pallidipes* which further complicates the tsetse eradication campaigns because the mass-reared colonies are normally established from flies collected from the target wild populations. Due to this negative effect, it is necessary to develop virus management strategies to enable sustenance of a healthy and productive colony size for the fly eradication campaigns. Designing a strategy that would interrupt the replication/transmission cycle of the virus in the colonies requires a comprehensive understanding of the mechanism involving the vector-virus interactions. The aim of our study was to establish an extensive protein map of *G. pallidipes* salivary gland secretome proteins and SGHV proteins in hypertrophied salivary glands, using stringent mass spectrometry criteria to validate the potential proteins, and to establish possible *Glossina*-SGHV interactions. The substantial differences between the protein profiles of the secretomes of hypertrophied and non-hypertrophied salivary glands, and the analysis of the nanoLC-MS/MS-supported secretome proteins data obtained demonstrate that a large proportion of the proteins identified are indeed secreted.

The data presented in this study demonstrates that the *G. pallidipes* salivary gland secretome encompasses a wide spectrum of proteins that may be required for the different facets of the SGHV infection cycle from viral attachment to egress of the virions from infected *Glossina* cells. On the basis of our previous proteomic analysis of GpSGHV virions [28] and the data presented in this study, some interactions between *Glossina* and SGHV proteins can be predicted (Table 3). It is to be noted that the *per os* infectivity factors (PIFs) were only detected in very low abundance, and although they are not included in our final pool of the salivary proteins, they probably play a vital role in the oral infection of *Glossina* sp. One other SGHV protein that was noted to be present in very low abundance was the baculovirus ODV-E66 (PIF4) homolog. This protein is known to be involved in the initial attachment of the baculovirus in the mid-gut epithelia of infected insects, and it is likely to play a similar role in the case of *Glossina*.

**Table 3:** Putative *Glossina*- SGHV protein interactions.

*Glossina* secretome proteins	SGHV proteins(ORFs in brackets)	Putative *Glossina*-SGHV protein Interactions
C-Lectins, hsc70, hsp70 & 90, TEPs	*Per os* infectivity factors (**1, 102, 53, 76**), glutathione-S-transferase (**46**)	Viral entry & signalling
Actins, Rho GTPase, GAP, molecular chaperones, ARFs	Lecithin-cholesterol acyltransferase (**6**), casein kinase I-δ (**41**), spectrin (**10**)	Birectional cytoplasmic transport & docking at nuclear pore complex (NPC)
HSPs, Translation initiation factors, RNA-helicase	Thymidylate synthase (**35**), dihydrofolate reductase thymidylate synthase (**36**), HSP90-like ATPase (**39**), p53-transcription factor-like (**62**), ABC-ATPase (**64**)	Viral DNA transcription,replication and translation
NTPase-/Torsin-like, DnaJ/Hsp40, molecular chaperones, hsp70-4, ER-PDI	Pre-mRNA splicing factor (**49**), FAD-sulfhydryl oxidase (**72**), type-IIe restriction enzyme (**93**), chitinase-II (**27**), maltodextrin glycosyltransferase (**38**), transport-channel proteins (**85, 96**), vesicle-associated membrane protein (**97**)	Maturation & nuclear egress of mature virions from infected salivary gland cells

TEPs = thioester-containing proteins; hsc = heat shock cognate; hsp = heat shock protein; GAP = GTPase-activating protein; ARFs = ADP-ribosylation factor; ER-PDI = endoplasmic reticulum protein disulphide isomerase.

Summary of the putative interactions between *Glossina* and SGHV proteins during the different facets of the virus replication cycle. The SGHV ORFs encoding the respective viral proteins are indicated (bold and in brackets).

Taken together, the identification of putative host-viral protein interactions opens novel avenues for the development of mitigation strategies against GpSGHV infections. Such strategies could include immune-interventions whereby virus-specific antibodies against PIF proteins could be supplemented in the blood meals used in the membrane-feeding in tsetse production facilities. This would lead to immuno-complexion of SGHV virions in the blood meals, which would block the horizontal transmission of SGHV from fly to fly. In addition, phage display-selected gut epithelia-binding peptides such as derived from the ODV-e66 protein (PIF-4) homolog could be designed to impede the attachment of SGHV to the *Glossina* mid-gut and subsequent movement of the virus into the fly hemocoel. Such chemically-synthesized oligopeptides or the phage display library expressing the active peptides could be supplemented in the flies' blood meals and, thereby, upon ingestion of the meal, the peptides would out-compete the viral homologs (ODV-e66) in the attachment to the mid-gut receptors [156]. By this approach, vertical transmission of the virus from mother-to-offspring would be interrupted. Finally, SGHV-specific genes could also be targets for RNA interference (RNAi).

### Future perspectives

The roles of *Glossina* and SGHV proteins identified in this study need to be experimentally established. Still to be investigated are questions particularly with regard to *Glossina* specificity and how SGHV overcomes various transmission barriers in the tsetse fly. Also to be resolved are the roles played in *Glossina* specificity by SGHV proteins, *Glossina* proteins, virus receptors, *Glossina* symbionts, as well as the role of hemolymph and other tissues in the viral transmission process. In addition, the barriers to transovarial (vertical) transmission of SGHV in tsetse remain grossly under-investigated. We hope that with the cataloguing of *Glossina* salivary glands secretome proteins, the detection of SGHV proteins in hypertrophied salivary glands and the advent of new molecular technologies, that such roles can be elucidated further and eventually exploited to initiate novel strategies for controlling SGHV infections in tsetse mass rearing facilities.

## Supporting Information

Table S1 **Sixty-five salivary gland secretome proteins of** ***G. pallidipes*** **.** Sixty-five *G. pallidipes* salivary gland secretome proteins supported by nanoLC-MS/MS, confirmed by gene ontology (GO) annotation, presence of signal peptide sequences and by Blasts on the NCBI and *G. m. morsitans* databases.
(DOC)

Table S2 **Twenty-five SGHV-encoded proteins detected in the** ***G. pallidipes*** **hypertrophied salivary glands.**
(DOC)
