# Supplementary material for: The Salivary Secretome of the Tsetse Fly Glossina pallidipes (Diptera: Glossinidae) Infected by Salivary Gland Hypertrophy Virus
Source: PLoS Negl Trop Dis. 2011 Nov 22;5(11):e1371. doi: 10.1371/journal.pntd.0001371 (PMC3222630; doi:10.1371/journal.pntd.0001371)
Supplement: Table S1 — Sixty-five salivary gland secretome proteins of G. pallidipes . Sixty-five G. pallidipes salivary gland secretome proteins supported by nanoLC-MS/MS, confirmed by gene ontology (GO) annotation, presence of signal peptide sequences and by Blasts on the NCBI and G. m. morsitans databases. (DOC) [file pntd.0001371.s001.doc]

Supplementary Table 1: Sixty-five *G. pallidipes* salivary gland secretome proteins supported by nano LC-MS/MS, confirmed by gene ontology (GO) annotation, presence of signal peptide sequences and by Blast searches in the NCBI and *G. m. morsitans* databases.

| **Contig ID^a^** | **Accession No.** | **Protein Name** | **Mol. Mass (kDa)** | **Length (aa)** | **Unique peptides** | **Conserved domain** | **BLAST e-value** | **GO Functional Annotation** |
| --- | --- | --- | --- | --- | --- | --- | --- | --- |
| cn11113 | ADD18584.1 | SG growth factor 1 | 5.388 | 49 | 2 |  | 5e-10 |  |
| cn13111 | AAL87009.1 | Protease-Inhibitor | 8.6228 | 77 | 2 | SBPI/Kunitz | 2e-16 | MF: (endopeptidase inhibitor); CC: (extracellular; cell part) |
| cn699 | ADD20212.1 | Niemann-Pick (TypeC) | 17.307 | 151 | 6 | MD-2-related lipid-recognition | 1e-85 | BP: (mesoderm development) |
| cn128 | XP_002056444.1 | Peptidase-S1/S6 | 17.739 | 310 | 3 | Tryp_SPc | 2e-61 | BP: (melanization defense response); MF: (endopeptidase) |
| cn7404 | CBA11306.1 | Pheromone/Odorant-binding-protein-99b-precursor | 17.9 | 153 | 6 | PBP/GOBP | 4e-86 | MF: (odorant binding); BP: (salivary gland cell autophagic cell death); CC: (extracellular) |
| cn13435 | CBA11325.1 | General odorant-binding-protein | 17.923 | 154 | 2 | PBP/GOBP | 2e-76 | MF: (odorant binding); BP: (transport) |
| Gmm-2145 | ADD20435.1 | Hypothetical conserved protein | 18.229 | 162 | 2 | Metallophosphatase (MPP) & 5’-Nucleosidase | 4e-91 | BP: (nucleotide catabolism);MF: (hydrolase) |
| GMsg-6444 | ADD20479.1 | Nucleoside diphosphate kinase | 19.313 | 172 | 7 | NDPks | 1e-94 | BP: (DNA synthesis; cell division; adherens junction organization); MF: (SUMO binding); CC: (cytoplasm) |
| cn3611 | ADD20496.1 | Prostaglandin E2-synthase3/Hsp90 co-chaperone p23 | 20.951 | 180 | 5 | Α-crystallin-Hsp_p23-like | 5e-99 | Participate in Signaling by small GTPase |
| cn9015 | ADD18444.1 | Profilin protein chickadee | 23.823 | 210 | 2 | PROF | 2e-65 | BP: (adult lifespan determination; vitellogenesis;); MF: (Binds to actin- & membrane phosphoinositides; signaling in Ras pathway); CC(cytoplasm) |
| GMsg-8947 | ADD19876.1 | Secreted phosphatidyl-ethanolamine-binding protein | 24.441 | 217 | 4 | PEBP | 9e-121 | Serine proease inhibition; membrane biogenesis |
| cn4289 | ADD19264.1 | Cu^2+^Zn^2+^-superoide dismutase | 25.177 | 235 | 2 | SOD (P-class) | 5e-121 | MF: (superoxide dismutase) |
| cn7661 | CAP78961.1 | Antimiscobial peptide Attacin B | 26.581 | 250 | 2 | Attacin_N & C-terminal | 3e-111 | BP: (antibacterial humoral response);CC: (hemolymph) |
| Gmm-3045 | ADD19954.1 | Hypothetical protein | 26.589 | 230 | 3 |  | 3e-115 |  |
| Gmm-2613 | ADD19951.1 | Hypothetical protein | 26.784 | 230 | 6 |  | 6e-121 |  |
| cn15528 | ADD19811.1 | Ca^2+^-/Calmodulin-/Calcyphosine-like-protein | 26.992 | 242 | 5 | EF-hand, Ca^2+^ binding motif | 2e-118 | BP: (phagocytosis); MF: (ca^2+^-binding) |
| GMsg-7136 | ADD20155.1 | **Γ**-interferon inducible lysososmal thiol reductase | 27.011 | 243 | 2 | GILT | 1e-140 | Mediator of antiviral activity |
| cn9673 | ADD18879.1 | Salivary Antigen 5 precursor | 28.901 | 258 | 2 | SCP-like extracellular protein | 2e-150 | CC: (extracellular); MF: (endopeptidase inhibitor) |
| cn513 | ADD19989.1 | Salivary antigen 5-precursor variant | 28.909 | 259 | 17 | SCP_CRISP | 2e-123 | MF: (endopeptidase inhibitor); CC: (extracellular) |
| Gmm-3046 | ADD19043.1 | Tsal2 protein precursor | 29.127 | 253 | 4 | NUC | 7e-126 | MF: (nucleic acid binding; DNA/RNA non-specific endonuclase) |
| cn2771 | ABC48941.1 | Lipophorin | 30.01 | 275 | 4 | Lipoprotein (LPD_N) | 5e-160 | MF: (lipid transport)) |
| cn4297 | XP_002057612.1 | TEP2 protein precursor | 31.24 | 275 | 2 | α-2-macroglobulin (A2M_N) receptor | 6e-80 | MF: (endopeptidase inhibitor); CC: (extracellular region) |
| cn2281 | NP_523506.1 | TEP2 protein, IsoformA | 31.446 | 279 | 3 | ISOPREN_C2_like | 2e-115 | BP: (antibacterial humoral response); MF(endopeptidase inhibition) |
| cn9192 | ADD18265.1 | Hexamerin F1 (LSP-2) | 34.798 | 294 | 7 | Hemocyanin_N-& _M | 3e-149 | MF: (oxygen transport;nutrient reservoir);CC: (larval serum protein complex) |
| cn399 | ADD18704.1 | Serine protease inhibitor | 36.032 | 330 | 2 | Tryp_SPc | 9e-55 | MF: (endopeptidase); BP: (proteolysis) |
| cn6238 | ADD19820.1 | Trehalose-6-phosphate-synthas (component TPS1) | 37.979 | 338 | 2 | α-β-Haloacid Dehalogenase (HAD-SIF-IIB) | 5e-30 | MF: (catalase); BP: (trehalose synthesis) |
| cn2477 | CAQ53422.1 | NTPase-/Torsin-like-protein | 38.032 | 339 | 2 | Walker A &B motifs | 3e-101 | MF: (unfolded protein binding); BP: (ATP- & Mg^2+^-binding); CC: (ER) |
| GLAFD01TV | XP_002048727.1 | Quiescin sulfhydryl oxidase4 | 38.377 | 339 | 6 | PDO | 5e-76 | MF: (thiol-oxidase activity); BP: (cell redox homeostasis) |
| cn291 | ADD19085.1 | Major royal jelly protein protein | 38.685 | 342 | 3 | MRJP | 3e-53 | MF: (larval jelly protein;adult cuticle pigmentation); CC: (cellular component) |
| cn1577 | ADD18617.1 | Trypsin | 39.789 | 354 | 2 | Tryp_SPc | 7e-145 | MF: (endopeptidase); BP: (proteolysis) |
| cn8256 | ADD19420.1 | Fat body Chymotrypsin | 39.868 | 353 | 2 | Tryp_SPc | 3e-53 | MF: (endopeptidase); BP: (proteolysis) |
| GMsg-7644 | ADD19393.1 | Secreted angiopoietin-like protein | 39.95 | 350 | 2 | FReD | 1e-63 | MF: (receptor binding); BP: (signal transduction) |
| cn3048 | ADD18624.1 | DnaJ/Hsp40 protein | 40.013 | 354 | 5 | DnaJ-C | 3e-20 | BP: (protein (un)folding); MF: (hsp-binding; protein translation, translocation &degradation) |
| cn8409 | ADD18624.1 | Molecular chaperon | 42.936 | 386 | 7 | DnaJ | 2e-45 | MF: (REDOX;NAD/NADH-binding) |
| cn3041 | ADD18511.1 | Vacuolar ATPase sector accessory subunit-S1 Ac45 | 43.367 | 384 | 3 | ATP-synt_S1 | 1e-03 | MF: (rotational mechanism; V-type ATPase) |
| cn1049 | ABN58709.1 | Tsal2-protein-Isoform A | 43.992 | 388 | 11 | NUC | 2e-06 | MF: (nucleic acid binding; hydrolase; metal ion binding) |
| cn43 | Q2PQM7.1 | Chitinase-like-protein | 44.986 | 436 | 7 | GH18_Chitinase-like | 2e-147 | MF: (Chitin hydrolysis; imaginal disc growth factor); CC: (extracellular) |
| cn4273 | ADD18566.1 | Large serine protease | 46.844 | 423 | 3 | Tryp_SPc | 1e-54 | BF: (endopeptidase); BP: (proteolysis) |
| cn408 | ADD18797.1 | Calreticulin precursor | 46.94 | 406 | 5 | Calreticulin | 4e-99 | BP: (startle response; pupariation; molting); MF: (sequence-specific DNA binding; protein heterodimerization); CC: (ER; nucleus) |
| cn8210 | ABC25072.1 | Serine protease inhibitor-1 | 46.971 | 420 | 3 | SERPIN | 5e-99 | MF: (Chaperoning protein; endopeptidase inhibitor) |
| cn6032 | ADD19384.1 | Medium-chain-Specific-acyl-CoA dehydrogenase (mitochondrial) | 47.307 | 431 | 4 | ACAD/CaiA | 0e+00 | BP: (REDOX);MF: (acyl-CoA dehydrogenase); CC: (mitochondria) |
| cn8313 | ADD19747.1 | Yellow precursor | 47.51 | 415 | 5 | MJRP | 3e-98 | Control of adult cuticle & mouth part pigmentation |
| cn2784 | ABC25079.1 | Serine protease inhibitor | 48.605 | 437 | 9 | SERPIN | 3e-67 | MF: (endopeptidase inhibition) |
| cn3205 | ADD18309.1 | Chitinase–like protein-precursor | 51.254 | 459 | 17 | Glyco_hydro_18 | 8e-65 | BP: (chitin catabolism); MF: (cation binding); CC: (extracellular) |
| cn1718 | ADD18950.1 | Inorganic pyrophosphatase/nucleo-side remodelling factor subunit NURF3 | 51.403 | 453 | 3 | Pyrophosphatase | 2e-48 | BP: (ecdysone signaling); MF: (diphosphatase); CC: (cytoplasm) |
| cn2284 | ADD19233.1 | Yellow-f | 52.034 | 455 | 5 | MRJP | 2e-93 | BP: (melanin synthethesis); MF: (dopachrome isomerase); CC: (extracellular) |
| cn4622 | ABC25095.1 | Imaginal growth factor-3 | 52.469 | 468 | 2 | IDGF | 0e+00 | MF: (chitinase-related GH18); BP: (interaction with surface glycoproteins); CC: (extracellular) |
| cn8091 | ADD18562.1 | Translation Initiation factor 5C (eIF4-ᵧ/eIF5/eIF2-έ | 52.936 | 458 | 2 | W2 | 9e-16 | MF: (translation initiation factor binding); BP: (oogenesis);CC: (cytoplasm) |
| cn4758 | ABC25074.1 | Serine protease inhibitor-4 | 53.656 | 466 | 3 | SERPIN | 3e-92 | MF: (Toll signaling regulation; antifungal humoral response) |
| cn16500 | XP_002046796.1 | Hypothetical conserved protein | 53.731 | 461 | 3 | Glyco_hydrolase-16 | 2e-113 | BP: (innate immune response); MF: (bacterial cell surface binding) |
| GMsg-8203 | ABN80093.1 | Ecto-5‘-nucleotidase-related protein | 53.977 | 478 | 5 | MPP_CD-73_N | 1e-98 | MF: (hydrolase; nucleotide & metal binding);BP: (nucleotide catabolism) |
| cn13608 | Q2PQN0.1 | Chitinase-like protein Idgf1 | 54.435 | 475 | 2 | GH18_(IGDF1) chitinase-like | 6e-56 | MF: (Chitinase/ATP generation) ; BP: (Mn^2+^/Fe^2+^/ Zn^2+^- binding) ; CC: (extracellular) |
| Gmm-3154 | ADD18584.1 | Adenosine/AMP deaminase-related-growth factor C | 57.003 | 497 | 32 | Adm_rel/Metallo-dependent hydrolase | 4e-114 | BP: (purine biosynthesis); MF: (deaminase); CC: (extracellular space) |
| cn7518 | ADD20206.1 | Lectin | 57.87 | 487 | 9 | CLECT | 2e-97 | BF: (Recognition of PAMPs) |
| cn786 | ADD20271.1 | Protein disulfide isomerase | 59.046 | 521 | 5 | ER_PDI-(a,a’,b & b’ subfamilies) | 5e-37 | BP: (protein folding; REDOX homeostasis); CC(cell pole; ER lumen; cytoplasm) |
| cn3338 | ADD20489.1 | Aldehyde dehydrogenase | 59.145 | 546 | 7 | ALDH_F7/SF_AASADH/  δ-1pyr5 carbox2 | 1e-55 | MF(amino acid metabolism/oxidoreductase) |
| cn12530 | ADD20435.1 | 5'-nucleotidase-related-salivary protein | 61.603 | 551 | 7 | MPP_CD73_N | 7e-95 | MF: (hydrolase; nucleotide binding); BP: (nucleotide catabolism; Mn^2+^,Fe^2+^, or Zn^2+^- binding) |
| cn583 | ADD20425.1 | Carboxyesterase | 63.125 | 545 | 8 | COesterase_lipase | 2e-102 | MF: (hydrolase) |
| cn2587 | ADD18753.1 | Homogentisate 1,2-dixoygenase | 63.875 | 555 | 2 | HgmA | 0e+00 | BP: (REDOX); MF: (amino acid metabolism) |
| cn14986 | XP_002002462.1 | Angiotensin- converting enzyme | 69.118 | 594 | 7 | GluZincin/  Peptidase_M2 | 2e-170 | MF: (Zn-dependent metallopeptidase); BP: (sperm individualization); CC: (membrane; extracellular); |
| cn1859 | ADD19839.1 | Hypothetical conserved protein | 69.181 | 634 | 11 | XendoU | 2e-52 | BF: (Endoribonuclease -produces products with a 2‘-3‘ cyclic phosphate termini) |
| GMsg-8257 | ADD20246.1 | Heo1-protein | 71.965 | 624 | 6 | GH20 hexosamini-dase | 1e-132 | MF: (β-N-acetylglucosaminidase); BP: (CHO-metabolism); CC: (plasma membrane) |
| cn15340 | ADD20300.1 | Hsp70 cognate 3 | 72.657 | 657 | 5 | Hydantoinase/  Oxoprolinase | 9e-03 | BP: (sleep & heat response); MF: (ATPase); CC: (ER) |
| cn747 | XP_002002903.1 | Peptidase family M2 Angiotensin converting enzyme-related-protein-precursor | 73.179 | 629 | 2 | M2_AC | 0e+00 | BP: (proteolysis); MF: (metallopeptidase); CC: (membrane) |
| cn270 | AAM46784.3 | Transferrin | 75.362 | 335 | 12 | Transferrin | 2e-63 | BP: (defense response); MF: (transmembrane transport); CC: (extracellular) |

MF = molecular function; BP = Biological process; CC = Cellular function; TM = (hydrophobic) transmembrane domain; SP = signal peptide, GO = Gene Ontology;

^a^Contig ID obtained from the *G. m. morsitans* SGs expressed sequence tags (ESTs) library available from the International Glossina Genome Initiative (IGGI) (<http://old.genedb.org/genedb/glossina/>).
