# Supplementary material for: The Salivary Secretome of the Tsetse Fly Glossina pallidipes (Diptera: Glossinidae) Infected by Salivary Gland Hypertrophy Virus
Source: PLoS Negl Trop Dis. 2011 Nov 22;5(11):e1371. doi: 10.1371/journal.pntd.0001371 (PMC3222630; doi:10.1371/journal.pntd.0001371)
Supplement: Table S2 — Twenty-five SGHV-encoded proteins detected in the G. pallidipes hypertrophied salivary glands. (DOC) [file pntd.0001371.s002.doc]

Supplementary Table 2. Twenty-five SGHV-encoded proteins detected in the G. pallidipes hypertrophied salivary glands.

| **SGHV protein** | **Gene ID** | **Class** | **Mol. Wt (kDa)** | **Length (aa)** | **Putative Protein Name** | **Signature domain** | **Functional annotation/characteristics** | **Reference** |
| --- | --- | --- | --- | --- | --- | --- | --- | --- |
| SGHV006 | 5950830 | VP | 41.678 | 361 | Lecithin:cholesterol acyltransferase (LACT) | α/β hydrolase catalytic domain | Membrane-docking (to NPC); capsid processing (nucleocytoplasmic export) | [1-5] |
| SGHV010 | 5950892 | VP | 127.03 | 1104 | Spectrin | Spectrin repeats | Involved in cytoskeletal structure; anterograde protein trafficking | [6-8] |
| SGHV027 | 5950894 | NS | 53.064 | 458 | Chitinase-II | Chitinases family 18 active site (*O*-Glycosyl hydrolases) | Virus attachment to host cells and cell lysis | [9-11] |
| SGHV035 | 5950850 | NS | 10.424 | 87 | Thymidylate synthase | Thymidylate synthase Active site | Regulation of balanced supply of dNTPs during DNA replication | [12, 13] |
| SGHV036 | 5950826 | NS | 13.794 | 115 | deoxycytidylate hydroxymethylase (dCMP-HMase) | Thymidylate synthase, chain A | Viral DNA replication (pyrimidine metabolism; expressed “early” during viral infection) | [14, 15] |
| SGHV038 | 5950861 | NS | 136.55 | 1173 | Maltodextrin glycosyltransferase | α Amylsase catalytic domain (α-β barrel containing active site) | Possible involvement in the expression of receptor protein for transport across cell membrane | [16] |
| SGHV039 | 5950825 | VP | 37.659 | 335 | HSP90-like APTase (HATPase_C ) | TM*, SP; Gyrase, Hsp90, Histidine-Kinase, Mutl (GHKL) domain | Signaling; regulation of DNA supercoiling/(un) winding of DNA strands | [17] |
| SGHV041 | 5950839 | VP | 48.773 | 413 | Casein kinase isoform I-δ | Protein kinase domain | Establishment of directional movement of encapsidated viral genome by phosphorylating cytoskeletal components during viral entry and egress | [18, 19] |
| SGHV046 | 5950897 | VP | 61.536 | 533 | Glutathione S-transferase |  | Possible signaling (to circumvent host defense mechanism) |  |
| SGHV049 | 5950974 | NS | 10.009 | 90 | Pre-mRNA Splicing factor 9-like protein | WD40 repeat, G-β-repeat | PRP19-associated complex; Associates with spliceosome; Control production of ion-channel protein | [20] |
| SGHV050 | 5950959 | ? | 32.741 | 291 | Uncharacterized | α Helix-β stand-α helix |  |  |
| SGHV062 | 5950971 | NS | 512.12 | 4373 | p53 transcription factor-like | β-sandwich domain of Sec23/24 superfamily | Chromosome segregation (induced “early” in S-phase); p53 suppressor protein | [21-24] |

| SGHV064 | 5950966 | NS | 70.023 | 595 | ATP-binding cassette-type-(ABC ATPase) like protein | Zinc Finger domain | Translation initiation, ribosome biogenesis and capsid assembly | [25-27] |
| --- | --- | --- | --- | --- | --- | --- | --- | --- |
| SGHV067 | 5950859 | VP | 31.019 | 259 | Uncharacterized | α Helix-β stand-α helix |  |  |
| SGHV068 | 5950953 | VP | 12.651 | 108 | Riboflavin uptake protein, chain a (ECF Transporter) |  | Membrane-embedded (cellular component); binds to Riboflavin | [28] |
| SGHV069 | 5950843 | VP | 30.914 | 270 | Ca^2+^ and Zn^2+^ –binding protein | TM*, SP |  |  |
| SGHV072 | 5950835 | VP | 31.771 | 269 | FAD-dependent sulfhydryl oxidase | TM* | Involved in virion maturation (virion assembly; expressed “late” in infection cycle) | [29] |
| SGHV085 | 5950886 | VP | 30.09 | 255 | Signaling Protein | α Helix-β stand-α helix | Tyrosine kinase-dependent signaling;Transport of viral structural proteins | [30] |
| SGHV086 | 5950956 | ? | 70.176 | 592 | Uncharacterized | α Helix-β stand-α helix |  |  |
| SGHV093 | 5950912 | VP | 38.532 | 329 | Hydrolase (taype-IIe restriction enzyme) | TM* | DNA-binding & cleavage | [31] |
| SGHV094 | 5950827 | ? | 32.70 | 273 |  | α Helix-β stand-α helix |  |  |
| SGHV096 | 5950829 | VP | 43.527 | 381 | Metal-binding protein (transport channel protein) | Multiple- TM*, SP | Mn^2+^ & S_2_O_4_ ^2-^ -binding | [32] |
| SGHV097 | 5950855 | VP | 44.403 | 394 | Vesicle-associated membrane (exocytosis) protein | TM* | Involved in targeting and/or fusion of vesicles to target membrane | [33] |
| SGHV109 | 5950856 | VP? | 33.194 | 285 | Putative uncharacterized protein | Cystathionine-β-synthase (CBS) domain) |  | [34] |
| SGHV112 | 5950920 | NS | 19.057 | 167 | Regulatory protein | Helix-turn-helix | Association with PML nuclear bodies | [35] |

TM = (Hydrophobic) transmembrane domain; SP = Signal peptide; NS= Non-structural protein; VP= structural/capsid protein

* denote transmembrane domains of the SGHV proteins (external glycoproteins or transport channels) to anchor the proteins to the membrane by a hydrophobic domain.
